# Changes in hospital mortality for United States intensive care unit admissions from 1988 to 2012

**DOI:** 10.1186/cc12695

**Published:** 2013-04-27

**Authors:** Jack E Zimmerman, Andrew A Kramer, William A Knaus

**Affiliations:** 1 Department of Anesthesiology and Critical Care Medicine, George Washington University, 17612 Coatbridge Place, Olney, MD, 20832, USA; 2 Cerner Corporation, 1953 Gallows Rd., Suite 500, Vienna, VA, 22182, USA; 3 Center for Clinical and Research Informatics, North Shore Research Institute, 1001 University Place, Evanston, IL, 60201, USA

**Keywords:** intensive care unit, hospital mortality, time trends, post-acute care, patient discharge, severity of illness

## Abstract

**Introduction:**

A decrease in disease-specific mortality over the last twenty years has been reported for patients admitted to United States (US) hospitals, but data for intensive care patients are lacking. The aim of this study was to describe changes in hospital mortality and case-mix using clinical data for patients admitted to multiple US ICUs over the last 24 years.

**Methods:**

We carried out a retrospective time series analysis of hospital mortality using clinical data collected from 1988 to 2012. We also examined the impact of ICU admission diagnosis and other clinical characteristics on mortality over time. The potential impact of hospital discharge destination on mortality was also assessed using data from 2001 to 2012.

**Results:**

For 482,601 ICU admissions there was a 35% relative decrease in mortality from 1988 to 2012 despite an increase in age and severity of illness. This decrease varied greatly by diagnosis. Mortality fell by >60% for patients with chronic obstructive pulmonary disease, seizures and surgery for aortic dissection and subarachnoid hemorrhage. Mortality fell by 51% to 59% for six diagnoses, 41% to 50% for seven diagnoses, and 10% to 40% for seven diagnoses. The decrease in mortality from 2001 to 2012 was accompanied by an increase in discharge to post-acute care facilities and a decrease in discharge to home.

**Conclusions:**

Hospital mortality for patients admitted to US ICUs has decreased significantly over the past two decades despite an increase in the severity of illness. Decreases in mortality were diagnosis specific and appear attributable to improvements in the quality of care, but changes in discharge destination and other confounders may also be responsible.

## Introduction

Over the last two decades there has been a decrease in mortality for patients admitted to US hospitals. Among Medicare patients who may or may not have been admitted to an ICU, risk adjusted mortality fell by 18% to 46% between 1994 and 2007 [1,2]. Disease specific decreases in the 30-day mortality rate over time have also been reported for patients hospitalized for congestive heart failure (50%, 1993 to 2008) [3], community acquired pneumonia (28%, 1987 to 2005) [4] and surgery for subarachnoid hemorrhage (50%, 1980 to 2005)[5], coronary artery bypass graft (21%, 1999 to 2008) and other high-risk procedures [6]. Changes in hospital mortality have also been reported for US patients with disorders commonly managed in ICUs including sepsis (9.9%, 1979 to 2000) [7], acute lung injury (9%, 1996 to 2005) [8] and surgery for aortic dissection (17%, 1979 to 2003) [9].

To our knowledge temporal changes in hospital mortality have not been reported for patients admitted to US ICUs. In contrast, multi-institutional data have shown mortality reductions over time in Australia and New Zealand (4%, 1993 to 2003) [10] and England (13.4%, 1998 to 2006) [11]. Studies focused on ICU prognostic systems have also reported decreases in hospital mortality over the last two decades. These studies have repeatedly demonstrated 'model fade': the over prediction of mortality when risk adjustment models are applied to more recent data [12]. As a result, the Simplified Acute Physiology Score (SAPS) [13], Mortality Probability Model (MPM) [14], and Acute Physiology and Chronic Health Evaluation (APACHE) [15] have required repeated updating. The developers of these systems have attributed mortality over prediction to increased treatment effectiveness, improved care before ICU admission, and more frequent discharge to post-acute care facilities [13-15].

Patients who are admitted to ICUs and survive hospitalization have a high mortality rate in the six months after discharge [16]. Many of these post-discharge deaths are among patients transferred to other acute-care hospitals [17] or long-term acute care facilities [18,19]. These findings have raised concerns that hospital discharge practices may partially account for reductions in hospital mortality and adversely impact performance assessments that use predicted hospital mortality [17,20].

This study reports a time series analysis of hospital mortality among patients admitted to multiple US ICUs over a 24-year period. The objectives of the study are: to describe changes in mortality and case-mix among ICU admissions from 1988 to 2012; to describe mortality changes among ICU patients with specific diagnoses over the same period; and to present data about the potential impact of patient discharge destination on hospital mortality. Data comparisons used a Chi-square test for categorical measures and an analysis-of-variance test for continuous measures.

## Materials and methods

We performed a retrospective analysis using data obtained from ICUs that had installed an APACHE system. Data for patients admitted before 2001 were obtained from published studies of APACHE. This emanated from two time periods: patient data for 1988 to 1989 were obtained from institutions randomly selected to represent US hospitals [21] and for 1993 to 1996 from a nonrandomized cohort of US institutions [22]. For admissions from 2001 onwards data were obtained for individual patients in the electronic APACHE database and grouped into three-year intervals. Multiple Institutional Review Boards (George Washington University, University of Maryland, Baystate Medical Center) waived the need for informed consent, deeming the use of these databases to not be human subject research requiring approval [15,21-24].

Patient data were generated as a result of patient care and collected for consecutive unselected ICU admissions. Demographic and clinical data were recorded on ICU day one, and vital status at ICU and hospital discharge. We did not collect data for patients with burns, ICU stay <4 hours, age <16 years and excluded second or subsequent ICU admissions. Further details about data collection, variables and the reliability of APACHE data have been described elsewhere [15,21,22].

### Changes in mortality over time

We examined changes in hospital mortality for all eligible first ICU admissions from May 1988 to June 2012. Concurrently we tracked changes in mean age and the day 1 acute physiology score (APS) of APACHE III. We repeated these analyses for patients who were mechanically ventilated during ICU day 1. We did not compare standardized mortality ratios for each time interval due to the numerous updates made to the APACHE mortality prediction over this 24- year interval [15,22].

To account for differences in diagnostic coding during the study, we examined only diagnoses with a consistent definition across all time intervals. We excluded diagnoses with <10% mortality during 1988 to 1989 to eliminate diagnoses that might have a small absolute change in mortality, which appears as a large relative mortality difference. These criteria produced 22 diagnoses. Using mortality rates in 1988 to 1989 as the baseline, we calculated the percent change in mortality in 2010 to 2012 for the 22 consistently defined diagnoses, and grouped them according to the percent decline in mortality: >60%, 51% to 59%, 41% to 50%, and 10% to 40%. Age and severity of illness (APS) data were available in aggregate but not for individual patients in the 1988 to 1989 and 1993 to 1996 cohorts were not available, thus we could not track diagnosis-specific changes in these variables.

### Changes in discharge destination over time

For each ICU admission the discharge destination was recorded using a computerized pick list; this information was only available for admissions from 2001 to 2012. Discharge destinations included death, home (with or without home health care), another hospital (including long term acute care facilities), nursing home (including assisted living facilities), skilled nursing facility (including inpatient rehabilitation facilities) and other destinations. Discharge to a post-acute care (PAC) facility was defined by discharge to skilled nursing, inpatient rehabilitation or assisted living facilities [24,25].

To assess the potential impact of changes in discharge destination on hospital mortality we examined the percentage of patients discharged home for each 3-year time interval from 2001 to 2012. We then repeated this analysis for each of the 22 diagnoses described above. To eliminate large changes in discharge destination due to small sample size we excluded diagnoses with <2,500 admissions in 2010 to 2012, the interval with the smallest number of patients.

We did not perform statistical analysis of differences between time periods. Analysis was not performed because significance was assured by the large size of each cohort.

## Results

The number of hospitals, ICUs and patients included in each data collection interval from May 1988 to June 2012 are shown in Table 1. The total number of ICU admissions was 482,601 and each time interval included ≥40 hospitals and ≥40 ICUs. With the exception of 1988 to 1989, all intervals contained at least 35,000 first admissions. The characteristics of the hospitals and ICUs are shown in Table 2. In general, hospital bed-size, teaching status, and geographic region were well dispersed, except for a smaller proportion of hospitals in the Northeast. The modal type of ICU was mixed medical-surgical, but most unit types were represented across the study.

**Table 1:** Number of hospitals, ICUs, and admissions during each time period.

Time Interval	# Hospitals	# ICUS	# Admissions
1988 to 1989	40	42	17,440
1993 to 1996	161	285	37,668
2001 to 2003	71	166	110,534
2004 to 2006	57	136	101,031
2007 to 2009	98	199	113,743
2010 to 2012	90	180	102,225
		Total	482,641

**Table 2:** Characteristics of hospitals and ICUs in each time period

	**1988-1989**	**1993-1996**	**2001-2003**	**2004-2006**	**2007-2009**	**2010-2012**
	
# of hospitals	40	161	71	57	98	90
Region						
Northeast	17.5%	19.9%	4.2%	0.0%	16.0%	21.1%
Southeast	32.5%	29.2%	31.0%	31.6%	27.0%	21.1%
Midwest	30.0%	23.0%	35.2%	35.1%	33.0%	41.1%
West	20.0%	28.0%	29.6%	33.3%	24.0%	16.7%
Teaching Status						
COTH	52.5%	40.4%	16.9%	17.5%	34.0%	54.4%
Teach, non-COTH	10.0%	19.9%	36.6%	43.9%	37.0%	24.4%
Non-teach	37.5%	39.7%	46.5%	38.6%	29.0%	21.1%
Bedsize	474	464	453	501	497	473
# of intensive care units	42	285	166	136	199	180
ICU Type						
Mixed Medical-Surgical	71.0%	33.3%	36.5%	30.9%	31.5%	36.7%
Medical	10.0%	19.6%	12.6%	15.4%	19.8%	17.4%
Surgical	16.0%	22.1%	14.4%	17.7%	21.3%	22.9%
Other	3.0%	25.0%	36.5%	36.0%	27.4%	22.9%

COTH, Council of Teaching hospitals; Other, cardiothoracic, coronary, neurological, and trauma units

The characteristics of patients admitted from 1988 to 2012 are shown in Table 3. For each cohort, patient age and severity of illness (APS) increased over time. There was a declining trend in the percentage of patients who were admitted post-operatively, from floors or by direct admission. Conversely, an increasing percentage of patients were admitted from another hospital or a step down unit. During the overall study period ICU length of stay decreased by 23% and hospital length of stay decreased by 38%.

**Table 3:** Characteristics of ICU patients admitted during each time period.

Cohort	1988-1989	1993-1996	2001-2003	2004-2006	2007-2009	2010-2012
# Admissions	17,440	37,668	110,534	101,031	113,743	102,225
Age (mean)	59.3	59.6	61.9	61.4	61.3	61.4
APS (mean)	39.3	34.2	39.0	39.3	42.0	42.2
≥ 1 chronic health conditions	12.0%	13.3%	10.0%	14.9%	14.8%	14.0%
% on ventlator, day 1	33.9%	Not Available	39.6%	37.9%	39.7%	36.0%
Location before admission						
Post-operative	42.3%	30.3%	35.8%	34.7%	26.5%	23.1%
Emergency Room	35.5%	39.0%	33.3%	32.9%	37.7%	37.8%
Floor/Dir Adm/Other	16.4%	16.5%	12.9%	12.5%	14.6%	13.7%
Other hospital	2.4%	7.7%	8.8%	8.1%	12.3%	15.7%
ICU transfer	3.3%	3.7%	5.7%	7.1%	3.5%	2.0%
Step down unit	N/A	2.9%	3.6%	4.7%	5.4%	7.7%
% Emergency surgery	9.0%	6.4%	4.5%	5.5%	5.4%	4.9%
% Race = white	80.3%	78.0%	73.9%	75.8%	78.2%	79.2%
ICU LOS (mean days)	4.64	4.30	3.43	3.86	3.96	3.58
Hospital LOS (mean days)	15.6	11.6	10.0	10.9	10.8	9.7
Hospital Mortality %	17.3%	12.4%	12.6%	11.9%	12.1%	11.3%

APS, acute physiology score; LOS, length of stay.

### Changes in patient mortality

**Figure 1:**
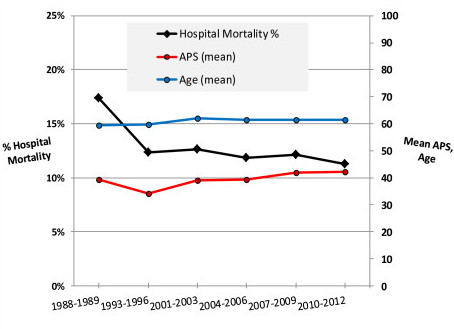
**Hospital mortality, age, and acute physiology score (APS) for 482,601 ICU admissions from 1988-1989 to 2010-2012**.

Temporal trends from the1988/1989 to the 2010/2012 time interval for aggregate mean hospital mortality, age, and APS for the 482,601 admissions are shown in Figure 1. There was a relative decrease in mean hospital mortality of 35% from 1988/1989 to 2010/2012 (*P *<0.001). Age increased slightly over this time period, but severity of illness (APS) increased by 7.4% (*P *<0.001). When stratifying the mortality trends by whether or not a patient was ventilated on ICU day 1, the changes in mortality were parallel between the two strata. These changes are shown as supplementary information [see Additional File 1, Figure S1].

**Figure 2:**
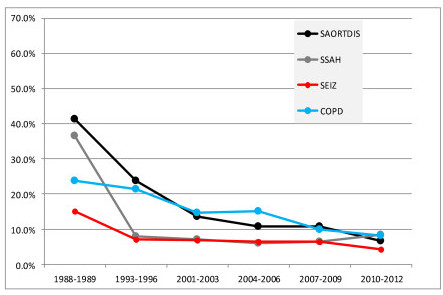
**Diagnostic groups with a >60% reduction in mortality from 1988-1989 to 2010-2012**. Definition of abbreviations: COPD, chronic obstructive pulmonary disease; SAORTDIS, surgery for aortic dissection; SEIZ, seizures; SSAH, surgery for subarachnoid hemorrhage.

**Figure 3:**
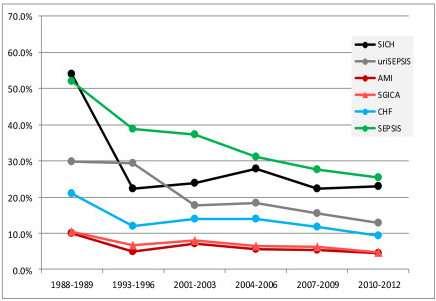
**Diagnostic groups with a 51% to 59% reduction in mortality from 1988-1989 to 2010-2012**. Definition of abbreviations: AMI, acute myocardial infarction; ; CHF, congestive heart failure; SEPSIS, sepsis, non-urinary tract; SGICA, surgery for gastrointestinal malignancy; SICH, surgery for intracerebral hemorrhage; uriSEPSIS, sepsis, urinary tract.

Table 4 shows the 22 ICU admission diagnoses that were consistently defined throughout the study and had >10% mortality during 1988/1989. There were 14 medical and 8 postoperative diagnoses. For 19 (90.5%) of the 22 diagnoses there was a >20% mortality decline from 1988/1989 to 2010/2012. The remaining three diagnoses had a 0 to 20% decline. Compared to the 1988/1989 baseline there was a >60% reduction in mortality for four diagnostic groups by 2010/2012 (Figure 2); they included surgery for aortic dissection (84%), surgery for subarachnoid hemorrhage (77%), seizures (72%), and chronic obstructive pulmonary disease (66%). There was a 51% to 59% mortality reduction for six diagnostic groups (Figure 3); they included surgery for intracerebral hemorrhage (58%), sepsis, urinary tract (57%), acute myocardial infarction (57%), surgery for gastrointestinal (GI) malignancy (57%), congestive heart failure (55%) and sepsis, non-urinary tract (51%).

**Table 4:** Diagnoses with a consistent definition and >10% mortality rate in 1988 - 1989

NON-OPERATIVE DIAGNOSES (number = 14)	POSTOPERATIVE DIAGNOSES (number = 8)
Acute respiratory distress syndrome	Surgery for GI bleeding
Chronic obstructive pulmonary disease	Surgery for GI obstruction
Pneumonia (viral)	Surgery for GI perforation
Cardiac arrest	Surgery for aortic dissection
Congestive heart failure	Surgery for intracranial hemorrhage
Sepsis, non-urinary tract	Surgery for subarachnoid hemorrhage
Sepsis, urinary tract	Surgery for multiple trauma, including the head
GI bleeding (varices)	Surgery for GI cancer
GI Bleeding (upper, non-variceal)	
Intracerebral hemorrhage	
Stroke/Cerebrovascular accident	
Head trauma with either chest, pelvis, or spine injury	
Seizures	
Acute myocardial infarction	

GI, gastrointestinal.

**Figure 4:**
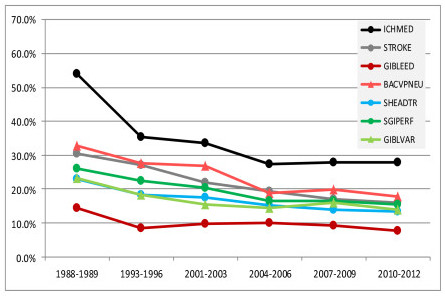
**Diagnostic groups with a 41% to 50% reduction in mortality from 1988-1989 to 2010-2012**. Definition of abbreviations: BACVPNEU, viral pneumonia; GIBLEED, gastrointestinal bleeding (upper); GIBLVAR, gastrointestinal bleeding, varices; ICHMED, intracerebral hemorrhage; SHEADTR, surgery for multiple trauma, including the head; STROKE, stroke/cerebral vascular accident.

**Figure 5:**
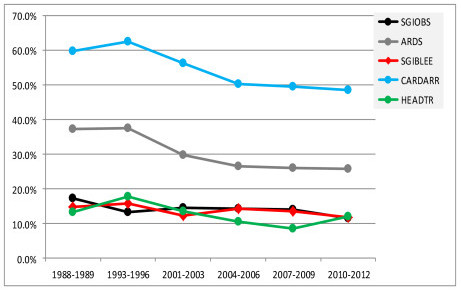
**Diagnostic groups with a 10% to 40% reduction in mortality from 1988-1989 to 2010-2012**. Definition of abbreviations: ARDS, acute respiratory distress syndrome; CARDARR, cardiac arrest; HEADTR, head trauma, with chest, abdomen, pelvis, or spine injury; SGIBLEE, surgery for gastrointestinal bleeding; SGIOBS, surgery for gastrointestinal obstruction.

There was a 41% to 50% mortality reduction for seven diagnostic groups from 1988/1989 to 2010/2012 (Figure 4); they included intracerebral hemorrhage (48%), stroke (47%), GI bleeding, upper-non-variceal (47%), viral pneumonia (46%), surgery for multiple-trauma, including the head (41%), surgery for GI perforation (41%) and GI bleeding from varices (41%). There was a 10% to 40% reduction for five diagnostic groups (Figure 5); they were surgery for GI obstruction (32%), acute respiratory distress syndrome (31%), surgery for GI bleeding (20%), cardiac arrest (19%) and head trauma with chest, abdomen, pelvis or spine injury (10%).

### Changes in hospital discharge destination

**Figure 6:**
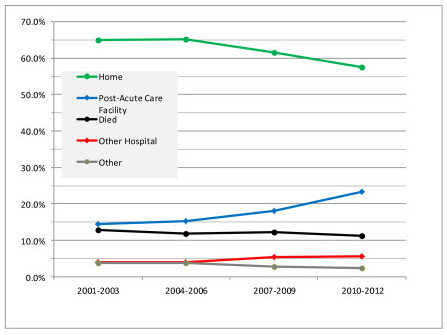
**Hospital mortality and discharge destination for 422,294 ICU admissions from 2001 to 2012**. Other hospital includes another acute care hospital or long term acute care facility.

Hospital death rates and the percentage of hospital discharges by destination for 427,533 ICU admissions from 2001 to 2012 are shown in Figure 6. Hospital death rates and the proportion of home discharges decreased over time. Conversely, the proportion of discharges to other hospitals and PAC facilities increased.

There were six diagnoses with sufficient sample size for analysis (>2,500 admissions in 2010 to 2012): cardiac arrest, congestive heart failure, GI bleeding (upper, non-variceal) stroke, acute myocardial infarction and sepsis (urinary tract). Changes in hospital discharge destination from 2001 to 2012 for patients with four of these diagnoses included an increased frequency of discharge to PAC facilities accompanied by a decreased hospital mortality rate and discharge to home. The exceptions were acute myocardial infarction (increase in discharge to home) and congestive heart failure (no change in discharge to home). These diagnosis specific results are shown graphically as supplementary information [see Additional File 1, Figures S2 to S7].

## Discussion

In a clinical database from multiple US ICUs, we found that mortality at hospital discharge had a relative decrease of 35% (17.3% to 12.4%) from 1988/1989 to 2010/2012. This mortality reduction was accompanied by an increase in severity of illness and number of chronic health conditions, factors that typically pose an increase in mortality risk. There was also a >50% mortality reduction across ten diagnostic categories over the same 24-year interval. Most of these dramatic relative decreases in hospital mortality rate occurred between 1988 to 1989 and 1993 to 1996. To the best of our knowledge, this is the first longitudinal analysis of hospital mortality rates in a clinical (non-administrative) database for a large number of admissions to multiple US ICUs.

The decrease in hospital mortality was not uniform across diagnostic categories. This may be because medical advances are typically related to the development of new therapies for specific diseases. The dramatic reductions in our study are consistent with prior reports of improved mortality for patients with stroke [1,2], acute myocardial infarction [1,2], congestive heart failure [1,3], chronic obstructive pulmonary disease [26] and sepsis [27]. Dramatic mortality reductions have also been reported after surgery for aortic dissection [9] and subarachnoid hemorrhage [28]. It is not our intention, however, to compare these disease specific mortality reductions with prior reports or relate them to new therapies.

Compared to reports from ICUs in other countries the changes in demographics and severity of illness were similar but reductions in hospital mortality were larger for these US ICU patients [10,11). Unfortunately, documented changes in the accuracy of APACHE mortality predictions over time [15,22] precluded evaluation of the impact of case-mix on changes in observed versus predicted mortality rates between 1988 and 2012. Our study's retrospective design, differences in patient selection across time periods, lack of information about ICU structure, process and use of disease-specific treatments, also make it impossible to assess the reasons for and timing of these mortality decreases. We are, therefore, unable to comment on the impact of differing ICU types, staffing by critical care specialists, ventilator and sepsis bundles, and changes in disease-specific therapy on mortality.

We are unable to account for the impact of Medicare's prospective payment system in reducing mortality at hospital discharge. This legislation created a substantial shift in the time US patients spent in acute care hospitals to PAC facilities between 1984 and 2002 [25,29]. These changes in discharge destination have previously been reported to shift hospital deaths to PACs [17,20], long-term acute care facilities [17,18] or other acute care hospitals [17,19]. Because we lacked data about discharge destination before 2001 we are unable to assess the impact of discharge destination on the dramatic fall in mortality between 1988 to 1989 and 1993 to 1996. Reductions in mortality might also be related to the 1998 Joint Commission performance measurement requirements for patients with acute myocardial infarction, heart failure, and pneumonia and subsequent public reporting in 2004 [30].

The modest reduction in hospital mortality (10.5%) from 2001 to 2012 may have been influenced, at least in part, by the increasing frequency of discharge to PACs and other hospitals (Figure 6). It is also possible, however, that severely ill patients discharged home in 2001 to 2003 might be going to PACs in 2010 to 2012 and, thus, not confounding the reduction in mortality. This emphasizes the need to obtain long-term survival data to properly assess temporal changes in mortality for patients surviving to hospital discharge [16-18].

The reduction in hospital mortality over this 24-year time interval explains why APACHE III (developed using 1988 to 1989 data) and APACHE IV (developed using 2002 to 2003 data) and other prognostic systems over predict mortality for contemporary ICU admissions. The over prediction of mortality is even greater for patients with specific diagnoses that had the largest mortality reductions between 1988/1989 and 2010/2012. The magnitude of these disease-specific changes may explain why recalibrating models that do not include specific diagnostic information often fails to improve calibration when tested in new populations [31-33].

Our study is subject to several limitations. First, our data are not necessarily representative of all US ICUs because they were obtained only from units with an APACHE system. However, there was great diversity in hospital size, teaching status, region, as well as ICU type. Second, our data are subject to a selection bias because data from 1988 to 1989 and 1993 to 1996 were population based, whereas data from 2001 onwards was from a self-selected sample of ICUs. In addition, the data for this study were not serially collected from the same hospitals over the 24-year interval, and we cannot exclude the possibility that changes in outcome were related to ICU based differences in quality of care. Third, we lacked individual patient data for 1988 to 1989 and 1993 to 1996 admissions. For specific diagnostic groups, this hampered our ability to evaluate the impact of changes in severity of illness and other confounders on mortality. Finally, we did not have information about mortality after hospital discharge. For this reason we were unable to evaluate accurately the impact of changes in discharge destination on hospital mortality.

## Conclusions

Hospital mortality for patients admitted to intensive care units has decreased significantly over the past quarter century despite an increase in the severity of patient illness. Decreases in mortality are diagnosis-specific and might be, in part, attributable to improvements in quality of care, but changing patterns in discharge destination and other confounders may also be responsible.

## Key messages

Hospital mortality for patients admitted to US intensive care units had a relative decrease of 35% between 1988 and 2012.

Reductions in hospital mortality were greater and more varied for patients within specific diagnostic groups.

Changes in hospital mortality over time explain why prognostic scoring systems over predict mortality when applied to more contemporary patient data.

Long-term outcome data are needed to assess the impact of discharge to post-acute care facilities on hospital mortality rates.

## Abbreviations

APACHE: Acute Physiology and Chronic Health Evaluation; APS: Acute Physiology Score; GI: gastrointestinal; MPM: Mortality Probability Model; PAC: post-acute care; SAPS: Simplified Acute Physiology Score.

## Competing interests

JEZ is a paid consultant and receives research support from Cerner Corporation. AAK is a full-time employee and stockholder in Cerner Corporation. WAK declares that he has no competing interests. Cerner Corporation, Kansas City, MO, supported the study. Cerner markets the APACHE Outcomes Clinical Information System and owns databases used for this study.

## Authors' Contributions

All authors took part in the planning, design, interpretation and drafting of the study. AAK acquired the data and performed the analyses. All authors have read and approved the manuscript for publication.

## Supplementary Material

Additional file 1 **Online supplement**. Description: Seven additional figures. One figure shows mortality over time stratified by whether or not a patient was ventilated. The remaining six figures show discharge destination over time for selected diagnoses. Click here for file
