# Supplementary material for: Changes in hospital mortality for United States intensive care unit admissions from 1988 to 2012
Source: Crit Care. 2013 Apr 27;17(2):R81. doi: 10.1186/cc12695 (PMC4057290; doi:10.1186/cc12695)

E. Figure1. Hospital mortality for 44,973 ICU admissions from 1988-1989 to 2010-2012, stratified by whether or not a patient received mechanical ventilation (MV) during day 1. Data on ventilator status for admissions during 1993-1996 were not available.

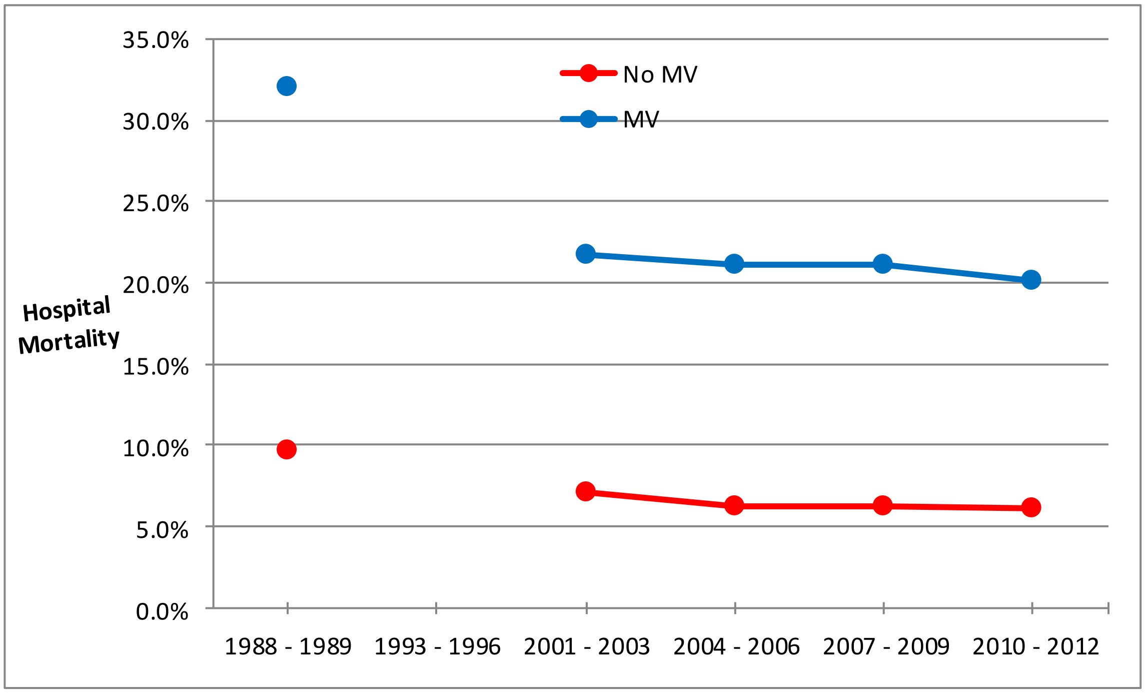

E. Figure 2. Discharge destination from 2001 - 2012 for patients with at least 2,500 admissions in 2010-12: Diagnosis of ***Cardiac arrest***

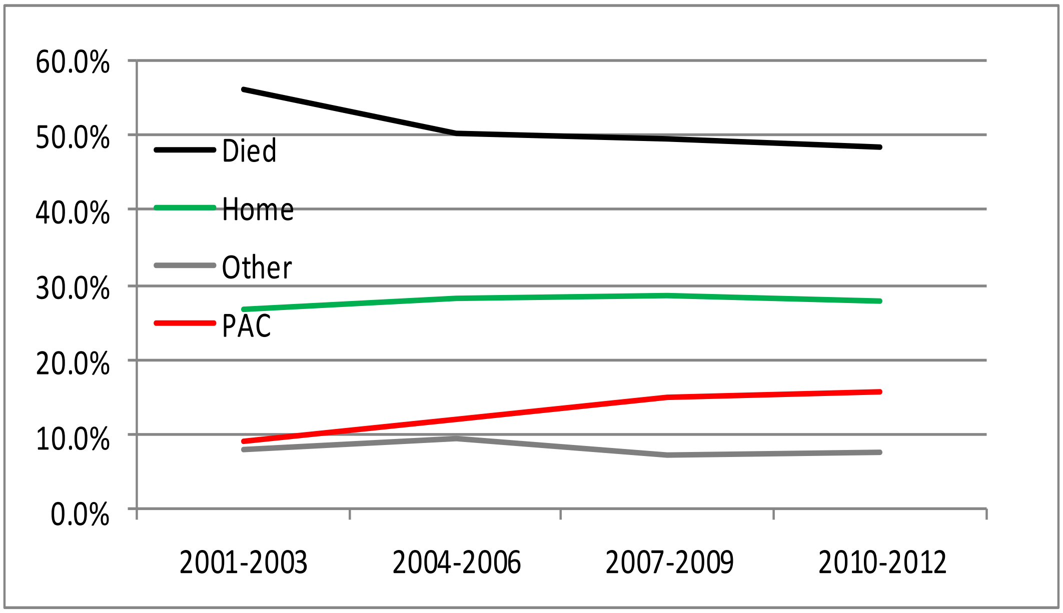

E. Figure 3. Discharge destination from 2001 - 2012 for patients with at least 2,500 admissions in 2010-12: Diagnosis of ***Congestive heart failure***

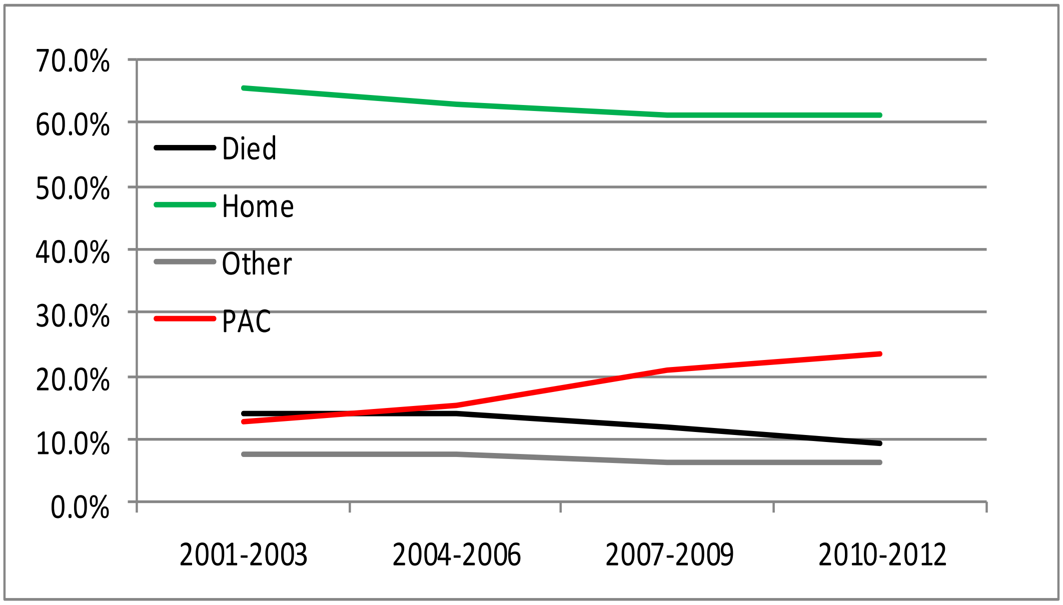

E. Figure 4. Discharge destination from 2001 - 2012 for patients with at least 2,500 admissions in 2010-12: Diagnosis of ***GI bleeding, upper***

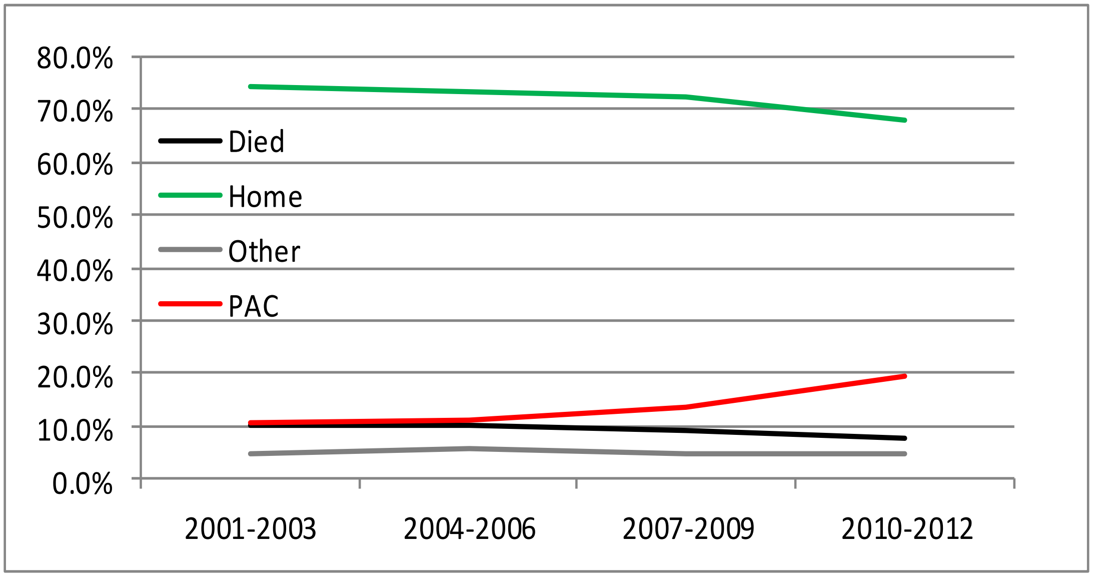

E. Figure 5. Discharge destination from 2001 - 2012 for patients with at least 2,500 admissions in 2010-12: Diagnosis of ***Stroke***

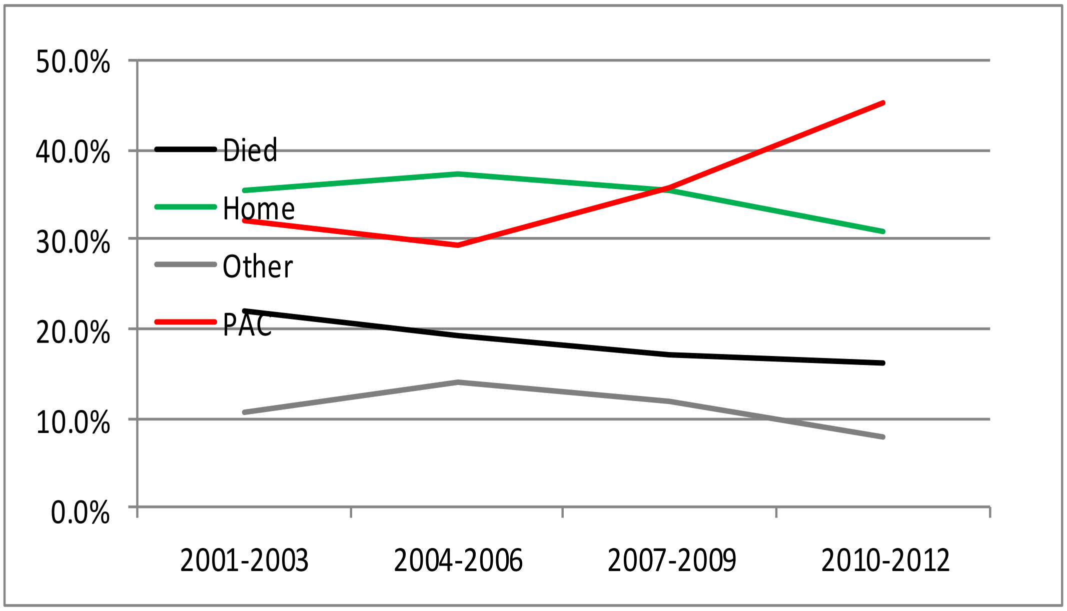

E. Figure 6. Discharge destination from 2001 - 2012 for patients with at least 2,500 admissions in 2010-12: Diagnosis of ***Acute myocardial infarction***

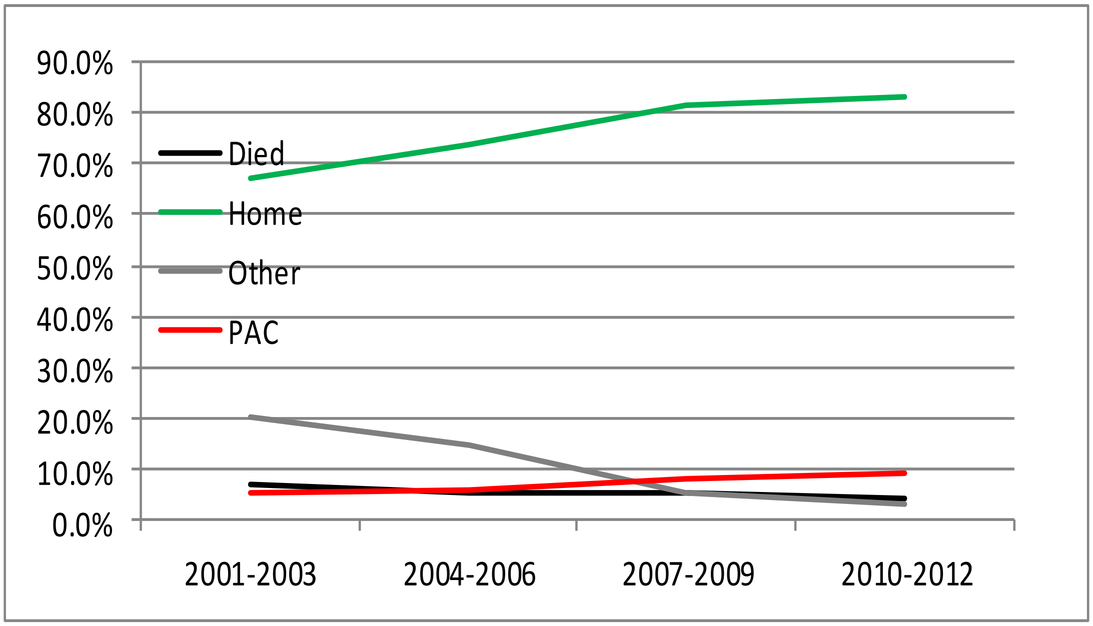

E. Figure 7. Discharge destination from 2001 - 2012 for patients with at least 2,500 admissions in 2010-12: Diagnosis of ***Sepsis***

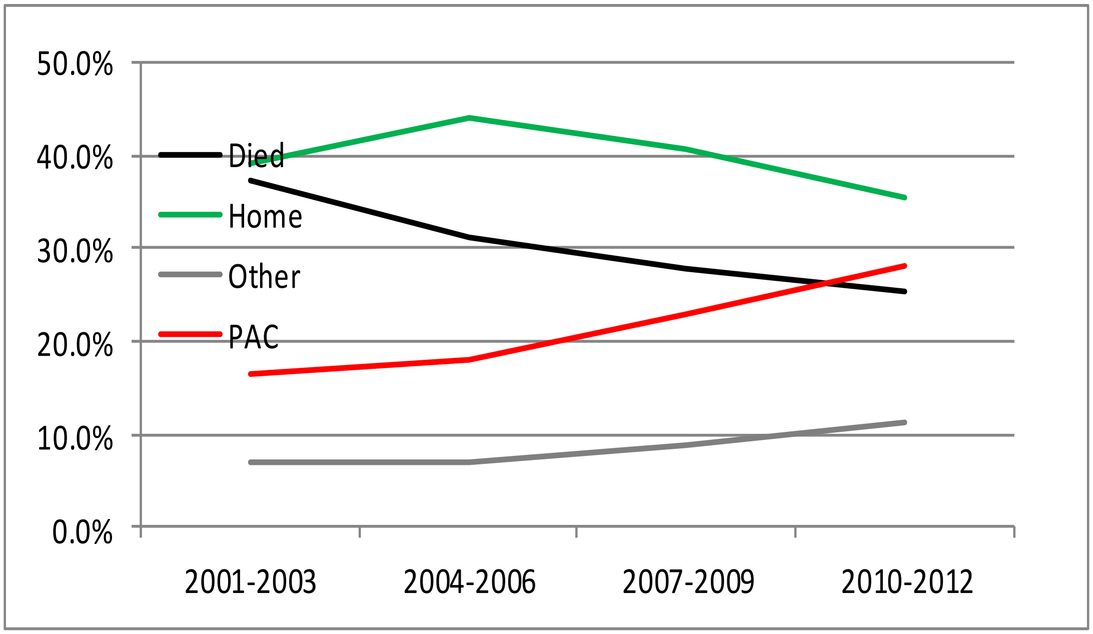

Supplement: Additional file 1 — Online supplement. Description: Seven additional figures. One figure shows mortality over time stratified by whether or not a patient was ventilated. The remaining six figures show discharge destination over time for selected diagnoses. [file cc12695-S1.PDF]
